# Variations in the viral hepatitis C prevalence and treatment status by material hardship and cumulative risk among people who inject drugs

**DOI:** 10.1016/j.dadr.2026.100468

**Published:** 2026-08-13

**Authors:** Sid S. Ganesh, Daniel R. Trigo, Rebecca P. Smeltzer, Cassandra Ward, Gilbert A. Orta Portillo, Karina Dominguez Gonzalez, Srehith Sannareddy, Bradley T. Conner, Ricky N. Bluthenthal

**Affiliations:** a Department of Population and Public Health Sciences, Keck School of Medicine, University of Southern California, Los Angeles, CA, USA; b Department of Psychology, Colorado State University, Fort Collins, CO, USA

**Keywords:** HCV, Material hardship, Immiseration, People who inject drugs, Harm reduction

## Abstract

People who inject drugs (PWID) are disproportionately impacted by viral hepatitis C (HCV). Among PWID, homelessness, poverty, and material insecurity elevate infectious disease risk. We hypothesized a positive association between material hardship (difficulty accessing basic needs) and lifetime diagnosis of HCV, and a negative association between cumulative risk (material hardship and years since first injection) and HCV treatment. From 2021–22, we conducted a survey among community-recruited PWID that included items on HCV outcomes, material hardship (a sum score of usually (4) to never (1) having difficulty finding food, clothing, shelter, restrooms, and showers in the past 3 months), and years since injection drug use initiation. We developed a latent variable, cumulative risk, by combining individual scores of material hardship indicators and years since first injection drug use. Among our sample of PWID (n = 471), 246 (53%) participants reported lifetime HCV diagnosis and 27% of those who tested positive for HCV reported ever or current treatment (n = 67). In modified-Poisson regression, a one-unit increase in material hardship score was associated with a 3% (OR: 1.03, 95% CI: 1.01%, 1.05%) increase in odds of HCV diagnosis. In latent modeling, among PWID testing positive for HCV, a one-unit increase in cumulative risk score was associated with a 0.28 (OR: −0.28, 95% CI: −0.50, −0.06), decrease in the Z-score odds of receiving HCV treatment. Findings emphasize structural interventions to strengthen material security and co-delivering basic needs with health services to improve HCV-related outcomes among PWID.

## Highlights

• Increase in material hardship was associated with increase in odds of HCV diagnosis.
• Increase in cumulative risk was associated with decrease in odds of HCV treatment.
• Findings support structural interventions **to** strengthen material security.

## 1 Introduction

### 1.1 Prevalence of hepatitis C virus globally and among people who inject drugs

Hepatitis C is a bloodborne virus that causes acute liver inflammation and can lead to chronic disease including cirrhosis, liver cancer, and hepatitis-related mortality (WHO). Hepatitis C virus (HCV) infection is a global epidemic (Bai et al., 2025), and 242,000 people died from HCV in 2022 (WHO, 2024). According to the World Health Organization (WHO, 2024), approximately 50 million people across the globe live with chronic HCV infection and one million new infections occur each year. Preventing, identifying, and treating HCV infection is a global public health priority. The WHO’s Global Health Sector Strategy on HIV, Viral Hepatitis, and Sexually Transmitted Infections (2022–2030) aims to end the viral hepatitis epidemic through reductions in new infections, particularly HCV infection, by 90%, and hepatitis-related deaths by 65% by 2030 (WHO, 2022).

One of the most common risk factors for preventable HCV infection both globally and in the United States is injection drug use (WHO, 2022; Degenhardt et al., 2017; Degenhardt et al., 2023). Worldwide, HCV prevalence among people who inject drugs (PWID) is over 50% (Degenhardt et al., 2017). In the United States, injection drug use was the most common risk factor for acute HCV infection in 2023, with 43% of cases with complete risk factor and exposure data reported to the Centers for Disease Control and Prevention occurring among PWID (Centers for Disease Control and Prevention, 2025). Behavioral risk factors for HCV transmission among PWID include long injection trajectories (i.e., number of years since injection; Hagan et al., 2008), receptive and distributive syringe sharing, sharing other injection materials and equipment (i.e., cookers, cotton, or water), unprotected sexual practices, and co-infection with HIV (Degenhardt et al., 2023). Additionally, structural factors common among PWID that increase likelihood of these risk practices and HCV transmission include recent incarceration (est. 10–40% of PWID globally), homelessness (10–50% of PWID globally), and sex work (5–20% of PWID globally; Degenhardt et al., 2023). Structural vulnerabilities – including homelessness, criminal-legal involvement, poverty, and lack of access to basic needs (material hardship)– reorganize environments to heighten infectious disease risk for PWID, including the risk of HCV transmission (Arum et al., 2021, Goldshear et al., 2021, Quesada et al., 2011, Flath et al., 2025). Material hardship, characterized by unstable and inconsistent access to food, clothing, shelter, showers, and restrooms, may intensify these inequities by reducing an individual’s capacity to engage in routine prevention, screening, and treatment (Odoms-Young et al., 2024). Material hardship has been associated with negative health outcomes among PWID including non-fatal overdose and withdrawal symptoms (Goldshear et al., 2025).

Untreated or undertreated HCV can impose a significant financial burden on healthcare systems. Current treatments for HCV rely on highly effective direct-acting antivirals (DAAs), which cure more than 95% of people in 8–12 weeks (Spengler, 2018). Without treatment, chronic HCV can lead to advanced liver cirrhosis, liver cancer, or end-stage liver disease, often requiring a liver transplant (Casey et al., 2023). In contrast, DAA therapy can drastically reduce these costs, reducing the cumulative cost burden per-patient from $199,500 to $57,000 for HCV-related conditions (Cline et al., 2021). Nationally, preventive DAA therapy to prevent late-stage liver disease translates into an estimated $49.2 billion in savings nationally over the course of a decade (Cline et al., 2021).

### 1.2 Disparities in accessing HCV treatment

Despite advances in the efficacy of treatment, HCV treatment utilization is lower among PWID compared to people who do not use drugs (Falade-Nwulia et al., 2020, Madden et al., 2018). Rates of treatment utilization among PWID averaged 23% compared to 65% among the general population (Falade-Nwulia et al., 2020, Nguyen et al., 2022). Overall, low treatment engagement and utilization among PWID contributes to ongoing transmission and increases the likelihood of long-term health complications (Degenhardt et al., 2023). Individual-level barriers to accessing HCV treatment include the absence of symptoms, perceptions that HCV is untreatable, stigma and discrimination, and limited awareness of available treatments (Morris et al., 2020). Systemic factors attributed to low HCV treatment utilization include physician perceptions of low adherence to treatment and reluctance to prescribe DAAs to PWID (Park et al., 2023, Amoako et al., 2021). However, systematic reviews and meta-analyses found that among those initiated on DAAs, PWID had similar rates of adherence to treatment and sustained virological response compared to those who did not inject drugs, particularly when treatment programs were tailored to PWID and integrated pharmacological substance use treatment (Latham et al., 2019, Aspinall et al., 2013).

Despite improvements to reduce barriers to HCV treatment engagement and utilization, such as the single daily dose and shorter treatment period (8 weeks) (Tsui et al., 2021) structural vulnerabilities, such as homelessness, poverty, low health literacy, and difficulty navigating healthcare systems, may further shape PWID ability to seek, engage with, and remain retained in HCV treatment (Arum et al., 2021). A recent prospective cohort study found that PWID had significantly higher proportion of postponed and missed treatment appointments, and that stable housing was significantly associated with treatment adherence among PWID (OR: 9.70, 95% CI: 2.10, 56.20), suggesting that unstable housing/homelessness may represent an important barrier to DAA adherence among PWID (Frankova et al., 2021). As biomedical interventions for HCV continue to advance, immiseration –socially and structurally driven material hardship–(Pfeiffer and Chapman, 2010) may undercut the efficacy of HCV treatment by hindering access and delivery. This study contributes to an important gap in the literature by examining associations between access to basic needs and lifetime HCV diagnosis and treatment among structurally vulnerable PWID.

### 1.3 Objective

Using self-reported data from a community-based sample of PWID recruited between 2021 and 2022, we analyzed how difficulties in accessing basic needs influenced HCV-related health outcomes. Despite advances in the efficacy of HCV treatment, HCV prevalence remains high among PWID, particularly those in community settings. To understand this gap, we examined the association between 1) material hardship (difficulty accessing basic needs) and HCV diagnosis, and 2) cumulative risk (material hardship and number of years since first injection drug use) and HCV treatment among PWID. Specifically, we hypothesized a positive association between material hardship and lifetime diagnosis of HCV, and a negative association between cumulative risk and HCV treatment.

## 2 Methods

### 2.1 Participants

We analyzed baseline survey data from a cohort of PWID (*N* = 472) in Los Angeles, California and Denver, Colorado between April 2021 to November 2022. Participants were recruited through convenience sampling from community-based sites, including syringe services programs, substance use disorder (SUD) clinics, and street outreach. Trained research staff administered 30–60-minute-long computer-assisted questionnaires. Inclusion criteria were 1) aged 18 years or older, 2) English fluency, 3) past 30-day self-reported injection drug use, and 4) past 30-day self-reported illicit opioid use. Survey domains relevant to this analysis included demographics*,* history of HCV diagnosis and treatment, and basic needs. Participants were compensated $20 for their time. This protocol was approved by the Institutional Review Board at the University of Southern California (HS−18–00624) and required informed consent from participants before interviews could be conducted.

### 2.2 Variables

#### 2.2.1 Predictor variables

##### 2.2.1.1 (a) Material Hardship (Model 1)

The predictor variable in Model 1 was material hardship in the past three months. Material hardship is a continuous index variable that aggregated responses to five validated questions representing difficulty accessing basic needs resources originally developed by Gelberg et al. (1997) as "competing needs.” Participants rated access to food, clothing, shelter, restrooms, and showers on a four-point scale from Never (1) to Usually (4). Scores from all responses were summed to develop the participant’s material hardship score. Material hardship scores ranged from 4 (not difficult to access food, clothing, shelter, restrooms and showers) to 20 (always difficult to access food, clothing, shelter, restrooms and showers).

##### 2.2.1.2 (b) Cumulative risk (Model 2)

In Model 2, we formed a latent construct that we refer to as *cumulative risk* indicated by each of the material hardship items and years since first injection use. We constructed this because long injection trajectories (i.e., number of years since injection) both increase risk for HCV, and contribute to health and social risk (Hagan et al., 2008). When combined with lack of access to basic needs resources, these risks accumulate but are not directly measurable. In a meta-regression of 72 studies on HCV prevalence in developed countries post−1995, Hagan et al. (2008) estimated that the mean prevalence of HCV among PWID was 32% (95% CI: 25.31, 39.58) after one year of injection initiation and increased to 53% at five years after injection initiation (95% CI: 40.69, 65.09). In this case, documented risks associated with injecting drugs combined with lack of access to basic needs may contribute to not receiving HCV treatment. In this way, *cumulative risk* is a latent construct that indirectly measures the accumulation of social and health-related risks over time. We specifically adjusted for cumulative risk in Model 2 as PWID who test positive for HCV are more likely to have long injection trajectories and more high risk injection practices (Zhou et al., 2019, Hagan et al., 2008, Hagan et al., 2004), both of which can lead to the accumulation of social and structural risks that contribute barriers to receiving treatment (Ford et al., 2025).

#### 2.2.2 Outcome variables

##### 2.2.2.1 (a) Lifetime HCV status (Model 1)

We asked all participants (N = 472), *“Have you ever tested positive for hepatitis C virus or HCV infection or been told by a health care professional that you are infected with the hepatitis C virus or HCV?”* We coded lifetime HCV status among all participants as ever testing positive or negative for HCV (0/1).

##### 2.2.2.2 (b) Treatment status (Model 2)

To the subsample of PWID who responded ‘yes’ (n = 248) to ever testing positive for HCV, we then asked, *“Have you ever or are you currently receiving treatment for hepatitis C virus or HCV infection?”.* Among those who tested positive for HCV, we coded treatment status as ever received/receiving treatment or never received treatment (0/1).

### 2.3 Covariates

#### 2.3.1 Lifetime HCV status (Model 1)

Model 1 was adjusted for sociodemographics, namely: number of years since initiating injection drug use (*years since first injection*), gender (male, female, transgender/other), race (White, Latinx, Black, Native, Asian, Pacific, Other), monthly income (less than $1000, $1000-$1400, $1401-$2100, $2100 or more) and by time-period of data collection, in six month increments (April to September 2021, October 2021 to March 2022, and April to December 2022). Model 1 co-variates were selected based on 1) the extant literature, 2) longer injection durations increasing HCV risk, and 3) ongoing transitions from injection to smoking within LA and Denver over the course of the study (Ganesh et al., 2025).

#### 2.3.2 Treatment status (Model 2)

We adjusted model 2 for sociodemographic variables, specifically monthly income and gender. We did not adjust this model for the number of years since first injection as the latent variable “cumulative risk” combined material hardship with the number of years since first injection. We also did not adjust this model for race and data collection period, as this sample was smaller and these were neither statistically nor theoretically indicated.

### 2.4 Analysis

#### 2.4.1 Lifetime HCV status (Model 1)

We conducted a modified Poisson analysis via the RQLM package in R (Noma, 2026, Zocchetti et al., 1995, Zou, 2004, Royall, 1986) to examine associations between material hardship and lifetime HCV status among all participants in the sample (N = 472), coded as ever testing positive or negative for HCV. We adjusted this model for years since first injection, gender, monthly income, race, and time period of study completion.

#### 2.4.2 Treatment status (Model 2)

Among a subsample of people who reported ever testing positive for HCV in their lifetime (N = 248) we conducted a latent probit regression analysis via the lavaan package in R (Rosseel, 2012) to examine associations between cumulative risk and treatment status. In a Latent Logistic Regression Model (Table 3a), we formed a latent measure termed “cumulative risk” composed of the individual latent indicators of material hardship (i.e., difficulty accessing food, shelter, clothing, showers, and restrooms; Gelberg et al., 1997) along with years since first injection (Hagan et al., 2008). For the latent model, we used thresholds of 0.90 for the Comparative Fit Index and Tucker Lewis Index to indicate good fit (Hu and Bentler, 1999). Table 3a provides information regarding probit coefficients for each latent indicator forming cumulative risk as a latent measure. We then assessed the final latent measure of cumulative risk as the main predictor of receiving HCV treatment while controlling for gender and monthly income (Table 3b).

## 3 Results

Out of 472 participants, one was excluded due to not providing information on HCV diagnosis, leading to a final overall sample of 471 participants. Among the 471 participants, the mean age was 41 years (SD=11.1) and most participants identified as male (77%), White (52%), and earning less than $1000 per month (53%). The majority of PWID (84%) were experiencing homelessness or unstable housing and the mean material hardship score was 9.47 (out of a maximum value of 20) indicating moderate difficulty finding food, shelter, clothing, showers and restrooms on average. A little over half the sample (53%, N = 248) tested positive for HCV, and of those who did test positive, only 27% (N = 67) were receiving treatment or had received HCV treatment at the time of the survey. Sociodemographic characteristics of the sample are available in Table 1.

**Table 1:** Demographic Characteristics of the Sample.

**Characteristic**	**Overall** N = 471^a^	**HCV Neg.** N = 223	**HCV Pos.** N = 248
	n(%) for categorical variables Mean (SD) for continuous		
**Age**	41 (11)	38 (10)	44 (12)
**Gender**			
Male	363 (77%)	178 (80%)	185 (75%)
Female	100 (21%)	39 (17%)	61 (25%)
Transgender	4 (0.8%)	3 (1.3%)	1 (0.4%)
Gender non-conforming	3 (0.6%)	3 (1.3%)	0 (0%)
Other	1 (0.2%)	0 (0%)	1 (0.4%)
**Race**			
White	245 (52%)	117 (52%)	128 (52%)
Latinx	123 (26%)	48 (22%)	75 (30%)
Black	26 (5.5%)	19 (8.5%)	7 (2.8%)
Native	47 (10.0%)	24 (11%)	23 (9.3%)
Asian	2 (0.4%)	2 (0.9%)	0 (0%)
Pacific	3 (0.6%)	1 (0.4%)	2 (0.8%)
Other	25 (5.3%)	12 (5.4%)	13 (5.2%)
**Monthly Income**^b^			
Less than $1000	249 (53%)	117 (53%)	132 (54%)
$1000 to $1400	87 (19%)	42 (19%)	45 (18%)
$1401 to $2100	61 (13%)	29 (13%)	32 (13%)
$2101 or more	70 (15%)	34 (15%)	36 (15%)
**Homeless or Unstably Housed (Last 3 Months)**			
No	77 (16%)	36 (16%)	41 (17%)
Yes	394 (84%)	187 (84%)	207 (83%)
**Material Hardship**^c^	11.8 (4.9)	11.5 (4.9)	12.0 (4.9)
**Receiving HCV Treatment**	67 (14%)	Not HCV positive	67 (27%)

a We have one missing value for HCV lifetime status.

b We have four missing values for monthly income.

c We have two missing values for material hardship.

### 3.1 Material hardship and lifetime HCV status (Model 1)

In a modified Poisson regression (Table 2), after controlling for years since first injection, gender, race, monthly income, and study completion timepoint, a one unit increase in the material hardship score was significantly associated with a 3 % (RR: 1.03, 95% CI: 1.01%, 1.05%) increase in the risk of being HCV positive.

**Table 2:** Modified Poisson Regression Model for Risk of HCV Positive Status (N = 471).

Variable	Risk Ratio	Standard Error	Confidence Limit (95%)	z	p-value
(Intercept)	0.17	0.29	0.10, 0.30	−6.06	0.00⁎
Material Hardship	1.03	0.01	1.01, 1.05	2.88	0.00⁎
Age	1.02	0.00	1.02, 1.03	6.02	0.00⁎
Gender (Ref: Male)	1.09	0.08	0.93, 1.26	1.07	0.28
Income	1.02	0.04	0.94, 1.10	0.47	0.64
Race	0.87	0.13	0.67, 1.12	−1.11	0.27
Time period (6 Month Increments)	0.93	0.06	0.82, 1.04	−1.32	0.19

⁎ Statistically significant at p < 0.05.

**Table 3a:** Latent Logistic Regression Model - Characterization of Cumulative Risk.

**Variable**	**Probit**	**95% CI**	**P-value**
Difficulty accessing food	-	-	-
Difficulty accessing clothing	1.05	0.624, 1.477	< 0.001
Difficulty accessing showers	1.15	0.627, 1.68	< 0.001
Difficulty accessing restrooms	1.15	0.606, 1.691	< 0.001
Difficulty accessing shelter	1.05	0.581, 1.511	< 0.001
Years since first injection	−6.39	−9.665, −3.114	< 0.001

^⁎^Statistically significant at p < 0.05

**Table 3b:** Latent Logistic Regression Model for Odds of Receiving HCV Treatment among People who Tested Positive for HCV (N = 248).

Variable	Probit	95% CI	P-value
Cumulative risk	−0.28	−0.507, −0.056	0.014
Gender	−0.23	−0.584, 0.121	0.198
Income	−0.02	−0.247, 0.063	0.245

^⁎^Statistically significant at p < 0.05.

### 3.2 Cumulative risk and treatment status (Model 2)

The latent model had a Comparative Fit Index of 0.934 and a Tucker Lewis Index of 0.947, indicating adequate fit. In the latent model, all of food (p < 0.001), clothing (p < 0.001), shower (p < 0.001), restroom (p < 0.001), and shelter access (p < 0.001) were positively associated with the latent measure of *cumulative risk*. In the final regression model, higher cumulative risk was associated with lower odds of accessing HCV treatment (p = 0.01) when controlling for income and gender. Among those with positive HCV status, a one point increase in cumulative risk was associated with a 0.28 decrease (OR: −0.28, 95% CI: –0.50, −0.06) in the Z-score odds of receiving HCV treatment, after adjusting for gender and income.

## 4 Discussion

In this study, over half of participants (n = 248, 52.5%) reported a lifetime positive HCV test, yet only 27% (n = 67) of those who tested positive for HCV had received treatment. Higher material hardship was associated with higher risk of positive HCV status. Similarly, among PWID with positive HCV status, higher cumulative risk was associated with lower odds of receiving treatment. Our findings are consistent with existing research, underscoring that material hardship may increase HCV infection risk and cumulative risk may reduce odds of receiving treatment (Valerio et al., 2024).

Our findings on material hardship and HCV risk underline the potential effectiveness of addressing immiseration structurally, institutionally, and via community-based initiatives to improve health outcomes. On a structural-level, safe, permanent housing, universal basic income programs, strengthening social services, and providing continuity of key programs including Supplemental Nutrition Assistance Program (SNAP), General Assistance/General Relief, are ways to address the harmful effects of immiseration, and reduce HCV risk (Gibson, 2024, Naumann et al., 2024, Lee et al., 2020, Madden et al., 2018). For example, rather than increasing the legalization and implementation of homeless encampment sweeps, structural interventions should prioritize permanent supportive housing for unsheltered PWID. Qualitative studies in California have documented how encampment sweeps interrupt substance use routines and lead to material loss of safer use supplies (e.g., syringes, cookers, cottons, sharps containers) and increase the likelihood of higher risk practices for HCV infection including rushed and more frequent injections as well as receptive syringe sharing (Goldshear and Kitonga, 2023, Chiang et al., 2022). Additionally, encampment sweeps result in the loss of medications and interfere with PWID’s ability to attend medical appointments, further disrupting treatment uptake and adherence (Goldshear and Kitonga, 2023, Mayer et al., 2024). Alternatively, unhoused people with HCV placed in permanent supportive housing in New York experienced significantly lower HCV-related morbidity and mortality including fewer liver-related emergency department visits and hospitalizations, and lower rates of all-cause and liver-related mortality (Miller-Archie et al., 2022).

At the institutional level, healthcare and service organizations can integrate an accessible care model that creates a low-threshold environment through flexible appointment scheduling and a supportive harm reduction framework. This approach can help clients identify their personal goals without pressuring them to adopt or reject any specific behavior. For example, routine opt-out HCV testing, needle and syringe programs and opioid substitution therapies have been associated with up to a 50% reduction in HCV acquisition among PWID (Platt et al., 2018). Further, integrating harm reduction programs within institutional settings such as safer syringe access within temporary and permanent shelter settings (Chayama et al., 2023), jails and prisons (Kronfli et al., 2025), as well as hospitals and emergency departments (Perera et al., 2022) can reduce risk of receptive syringe sharing and subsequent HCV infection. Expanding syringe access and coverage by directing funding to SSPs, expanding harm reduction vending machines, and conducting syringe exchange at jails, hospitals, community clinics, and overdose prevention programs are all evidence-based mechanisms for reducing HCV risk among structurally vulnerable PWID. Where possible integrating syringe exchange with basic needs access to co-deliver services may reduce the negative effects of material hardship and competing needs among indigent PWID. Embedding screening and treatment within harm reduction programs, shelters, and mobile clinics–paired with support in transportation, food assistance, and case management– could mitigate the structural barriers faced by materially deprived PWID and improve treatment initiation and completion (Dore, 2025).

At the community level, peer-led education, outreach, and culturally responsive prevention efforts can increase HCV awareness, reduce stigma, and connect individuals to harm reduction, testing, treatment, and supportive services. Together, these multilevel strategies can address both proximal risk behaviors and the structural determinants that sustain HCV transmission in populations at higher risk for HCV acquisition.

Our findings on cumulative risk and receiving HCV treatment have important implications for health service delivery stakeholders. Some ways to improve health service delivery include 1) identifying missed intervention points for linkage to HCV treatment in institutional (hospitals, upon release) and community settings (methadone clinics, drop-in centers, shelters, SSPs), and 2) delivery of HCV treatment alongside basic needs services (Schwarz et al., 2022). In the recent decades, both medicine and public health have been focused on advancing biomedical interventions, treatments, and health services. However, our findings are situated within a corpus of research indicating that structurally vulnerable PWID are unable to access existing biomedical interventions. For structurally vulnerable PWID, effective harm reduction interventions include a combination of low-threshold on-site HCV RNA testing and same day DAA initiation with services that address basic needs. These may include street medicine services, mobile syringe-exchange vans, mobile shelter clinics that also provide food, hygiene supplies, transportation vouchers, housing and income navigation aid, and peer case-management. Competing material needs and illness present cumulative risks to treatment seeking; co-delivering HCV treatment with material resources can reduce such barriers (Arum et al., 2021, Dore, 2025, Marshall et al., 2020, Valerio et al., 2024).

Time-sensitive interventions that reduce the burden of competing material needs may be of specific importance. For instance, randomized control trial evidence showcases the effectiveness of same-day initiation: the Seek, Test, and Rapid Treatment trial reported sustained virologic response at 12 weeks (SVR12) in 64% of young PWID receiving rapid on-site treatment compared to only 9.1% with facilitated referral (Eckhardt et al., 2022a). Similarly, the Accessible Care trial found that low-threshold on-site care at syringe service programs achieved intent-to-treat SVR12 among 67.1% of participants compared to 22.9% with standard referral, with those who initiated therapy achieving approximately 86% SVR12 in both arms, indicating improved engagement and treatment initiation (Eckhardt et al., 2022b). The mechanisms driving outcomes such as higher treatment initiation (Singh et al., 2023), linkage (Remy et al., 2019), and completion include: the removal of prior authorization, as it remains a significant barrier to HCV care (Javanbakht et al., 2020); contingency management (Norton et al., 2019) through medication blister packs and incentives; and the reduction of perceived stigma via peer educators (Batchelder et al., 2017).

These findings are relevant as they highlight a modifiable, intervention point i.e., access to basic needs resources, that may both reduce HCV risk and improve utilization and efficacy of existing evidence based biomedical treatments such as DAAs. Our findings point to the potential dual benefits (reducing risk and improving treatment access) of structural interventions aimed at reducing immiseration in the population.

### 4.1 Limitations

We report the following limitations. First, these data are the baseline data of a longitudinal, prospective cohort study that aimed to determine if changes in cannabis use frequency are associated with changes in frequency of opioid use and opioid-related health outcomes among opioid-using PWID in California and Colorado, states with legal medicinal and adult recreational use. Hence, the inclusion criteria was opioid use and injection drug use - so people were included if they were injecting simulants such as amphetamines. Second, this is a cross-sectional study and so we cannot make any causal inference. We recommend that future research examine these associations longitudinally to determine causality. Third, snowball sampling and respondent-driven sampling were not feasible, due to pandemic-related constraints. We recruited participants via convenience sampling from syringe service programs in Los Angeles and Denver as well as from community sites frequented by PWID such as parks and shower facilities (in Los Angeles). Recruiting PWID from community settings combined with convenience sampling likely resulted in a sample that may have a higher overall material hardship than the general population of PWID. However, here, our findings and analysis are specific to PWID who are socially and structurally vulnerable to negative health outcomes. Fourth, all data were self-reported, and we did not conduct chart reviews to verify participants’ medical histories related to HCV diagnosis or treatment. While the survey comprehensively covered medical history, there remains a possibility of recall bias or misreporting due to misunderstanding, omission, or miscommunication. However, self-report studies among PWID have shown negligible effects of recall bias (Dowling-Guyer et al., 1994). Lastly, we constructed model 2 as a latent model due to multi-collinearity between material difficulty and years since first injection. This, however, means that we asked participants about their lifetime HCV diagnostic status and treatment while only asking about recent material hardship in the last three months. This inherently precludes making causal conclusions as we cannot determine if HCV diagnosis occurred before or after conditions that define material hardship.

### 4.2 Conclusion

Our findings support the study hypothesis that immiseration is social determinant of elevated HCV infection risk and undermines access to treatment among PWID. In our community-based sample, each increment in material hardship was linked to significantly higher odds of a lifetime HCV diagnosis and lower odds of treatment engagement among those infected. These results highlight the necessity of structural interventions such as safe, permanent housing and universal basic income that strengthen access to basic needs for the population. Further, coupling antiviral scale-up interventions that alleviate basic needs with policies and programs that embed HCV screening and DAA delivery with harm reduction services, shelters, and mobile clinics, along with supports such as transportation, food assistance, and case management, are essential to overcoming structural barriers to accessing services among PWID.

## CRediT authorship contribution statement

**Sid S. Ganesh:** Writing – review & editing, Writing – original draft, Methodology, Investigation, Formal analysis. **Daniel R. Trigo:** Writing – review & editing, Writing – original draft, Formal analysis. **Rebecca P. Smeltzer:** Writing – review & editing, Writing – original draft, Formal analysis. **Cassandra Ward:** Writing – review & editing, Writing – original draft. **Gilbert A. Orta Portillo:** Writing – review & editing, Writing – original draft, Investigation. **Karina Dominguez Gonzalez:** Writing – review & editing, Writing – original draft, Project administration, Investigation. **Srehith Sannareddy:** Writing – review & editing, Investigation. **Bradley T. Conner:** Writing – review & editing, Writing – original draft, Formal analysis. **Ricky N. Bluthenthal:** Writing – review & editing, Writing – original draft, Supervision, Software, Resources, Project administration, Methodology, Investigation, Funding acquisition, Formal analysis, Data curation, Conceptualization.

## Ethics declaration

Written informed consent to take part in the study and to publish the article has been obtained from all participants or their legal representatives. The privacy rights of participants have been observed.

This study was performed in compliance with relevant laws, regulatory frameworks and guidelines where the research took place. This study was conducted in accordance with Institutional Review Board. This study was approved by the University of Southern California Institutional Review Board. (Approval No. HS−18–00624)

## Disclosure of interest statement

Sid S. Ganesh, Rebecca Smeltzer, Bradley T. Conner, Gilbert A. Orta Portillo, and Ricky N. Bluthenthal report financial support provided by National Institute of Drug Abuse (NIDA). Ricky N. Bluthenthal, Rebecca Smeltzer, Brad Conner, Jimi Huh, and Sid S. Ganesh were supported by NIDA R01-DA046049. Sid S. Ganesh is also supported by the Institute for Addiction Science pilot award PG1033682. This publication was also supported by SC CTSI (NIH/NCATS) through Grant UL1TR001855. Its contents are solely the responsibility of the authors and do not necessarily represent the official views of the NIH.

## Declaration of Competing Interest

The authors declare that they have no known competing financial interests or personal relationships that could have appeared to influence the work reported in this paper.
